# Priming effect of ^13^C-labelled wheat straw in no-tillage soil under drying and wetting cycles in the Loess Plateau of China

**DOI:** 10.1038/srep13826

**Published:** 2015-09-08

**Authors:** Enke Liu, Jianbo Wang, Yanqing Zhang, Denis A. Angers, Changrong Yan, Theib Oweis, Wenqing He, Qin Liu, Baoqing Chen

**Affiliations:** 1 Institute of Environment and Sustainable Development in Agriculture, Chinese Academy of Agricultural Sciences, Beijing, 100081, PR China; 2 Key Laboratory of Dryland Farming Agriculture, Ministry of Agriculture of the People’s Republic of China (MOA), Beijing, 100081, PR China; 3 Yanqing Service Center of Cropping Cultivation, Beijing, 102100, PR China; 4 Agriculture and Agri-Food Canada, Québec City, QC Canada G1V 2J3; 5 International Center for Agricultural Research in the Dry Areas, Damascus, Syrian Arab Republic

## Abstract

The objectives of this study were to determine the effects of drying and wetting (DW) cycles on soil organic carbon (SOC) mineralisation and on the priming effect (PE) induced by the addition of ^13^C-labelled wheat straw to long-term no-tillage (NT) and conventional-tillage (CT) soils. We observed that the SOC mineralisation rate in rewetted soils was greater than that in soils that were kept at constant water content. The proportion of CO_2_ derived from the straw declined dramatically during the first 10 days. The priming direction was first positive, and then became slightly negative. The PE was higher under DW cycles than under constant water content. There was no significant effect of the tillage system on the SOC mineralisation rate or PE. The data indicate that the DW cycles had a significant effect on the SOC mineralisation rate and on the PE, demonstrating a positive combined effect between wheat straw and moisture fluctuations. Further research is needed to study the role of microbial communities and C pools in affecting the SOC mineralisation response to DW cycles.

Soil organic carbon (SOC) mineralisation is controlled by multiple environmental factors, among which moisture may play a significant role^1^. Drying and wetting (DW) cycles can be defined as the repeated variations in soil water content from dry to wet states^2^. Natural DW cycles of soils, which are mainly caused by irrigation and the uneven distribution of rainfall throughout the year, are common and frequent events that profoundly affect SOC mineralisation^3^,^4^. Previous studies have shown that DW cycles induce inconsistent effects on SOC mineralisation. The majority of studies suggested that air-drying and subsequent rewetting induced less soil organic matter (SOM) mineralisation than constant water content situations^5^,^6^, while some have found the contrary^3^,^7^ and others found no significant changes^8^.

The ‘priming effect’ (PE) which can be defined as the change in the mineralisation of native soil organic matter (OM) due to the addition of new substrates, has been observed in many studies^9^,^10^. The addition of straw or other fresh organic matter to the soil can accelerate or retard SOC mineralisation, thus causing either positive or negative PE^11^,^12^,^13^,^14^. PE has been mainly studied in arable soils after the addition of plant residues under constant moisture^15^,^16^,^17^. However, little information is available on how PE can be affected by DW cycles.

Soil tillage is a management practice that modifies many soil properties and processes and therefore is susceptible to changes in PE intensity. Very few studies have investigated the long-term effect of no-tillage (NT) versus conventional tillage (CT) on PE. Studies have confirmed that soils under NT systems have an advantage over CT soils in maintaining and increasing surface SOC^18^,^19^. Changing tillage management can greatly modify chemical and microbiological properties that are able to generate variable PE intensity, particularly over the long term^20^.

The Loess Plateau of China, a semiarid region with water scarcity and large variations in inter-annual rainfall, is subject to frequent DW cycles and severe drought during the dry season. Therefore, this condition, coupled with frequent tillage and little straw return enhances the loss of carbon from agricultural soils^21^. Information on the effects of tillage management on SOC in the Loess Plateau already exists^22^,^23^,^24^. However, the PE-soil rewetting interactions are not well documented. Understanding how dry/wet cycles affect PE is important in predicting SOM dynamics, determining the effects of climate change on greenhouse gas (GHG) emissions from soils and improving soil C modelling. We suggest that differential PE intensities might occur under different tillage treatments, and this effect might help to explain the variations in SOM accumulation observed between different tillage systems. We also hypothesised that dry/wet cycles will influence the intensity and direction of the PE induced by wheat straw on NT and CT soils over time. To address these questions, we performed an incubation experiment in the laboratory over 120 days in which the NT and CT soils with or without winter wheat straw input were subjected to DW cycles or constant moisture. ^13^C-labelled winter wheat straw was used to distinguish between the CO_2_ from winter wheat straw mineralisation and that from SOC mineralisation. We aimed to quantify the effect of DW cycles on SOC mineralisation and on the intensity of the PE that was induced by winter wheat straw input and to determine the difference in the effects of CT and NT soils on SOC mineralisation.

## Methods

### Research site and soil preparation

The long-term experiment was established in 1992 and located in Linfen City, Shanxi Province, China (38° 6′ N, 113° 26′ E, 456 m asl). The average annual temperature is 10.7 °C with 180 frost-free days. This area of the Loess Plateau is characterised by a semiarid climate with a mean annual precipitation of 555 mm, falling mostly between July and September. The soils in the area are subject to frequent cycles of DW and severe drought during the dry season. The soil is a Cinnamon Loess, which is low in organic matter and slightly alkaline. Under the USDA soil classification system, the soils are defined as silt loam, and according to the FAO-UNESCO soil map^25^, the soil type is classified as Chromic Cambisol. The soil chemical properties are shown in Table 1. The basic physical and chemical properties of the soils (Table 1) and the methods used to derive them were previously reported by Liu *et al.* (2014)^22^, but the values are included here for easy reference and to aid in the interpretation of the findings.

At the beginning of the field experiment in 1992, the entire field was ploughed to a depth of 40 cm to mix the soil thoroughly and ensure uniform soil conditions in each experimental plot. The two tillage systems, conventional tillage and no-tillage with straw retained, were applied to the experimental plots from 1992 to 2012.

In the conventional tillage treatment, most of the winter wheat residues were manually removed, and only a small amount of standing stubble of approximately 8–15 cm in height remained on the soil. Mouldboard ploughing was performed to a depth of 15–20 cm after harvest during the first 10 days of June, and tine tillage was used for seedbed preparation in late September. During the fallow period (June to September), herbicide (2,4-D butylate) was applied at a rate of 0.9 kg ha^−1^ using a knapsack sprayer with a flat fan nozzle.

For the NT treatment, the winter wheat was harvested mechanically and standing stubble, approximately 15–20 cm in height was retained, with all wheat residues left as a mulch cover (average 3.75 t ha^−1^). During the fallow period, there was no tillage, and the weeds were controlled by herbicide only. The winter wheat was sown with a no-till planter between the 20^th^ and 30^th^ of September and harvested between the 1^st^ and 10^th^ of June.

Three replicates per treatment were carried out in randomised blocks, with plot sizes of 3 m × 80 m. The winter wheat variety used throughout the study was Linfen 225, planted at a seeding rate of 225 kg ha^−1^. The herbicide (2,4-D butylate) and insecticide (40% dimethoate) were applied at a rate of 0.9 and 0.3 kg (a.i.) ha^−1^, respectively, in April in the NT and CT treatments. Urea (CO(NH_2_)_2_), Diammonium phosphate ((NH_4_)_2_HPO_4_), and Potassium chloride (KCl) were applied as fertilisers to provide 150 kg N ha^−1^, 140 kg P_2_O_5_ ha^−1^ and 62 kg K_2_O ha^−1^. Each year all of the chemical fertilisers were applied in one dose to the top 20 cm of soil before sowing.

The soils were sampled from the 0–20 cm depth from the NT and CT fields in June 2012 after harvesting the winter wheat. After carefully removing the surface organic materials, fine roots and stone, each soil sample was air-dried and passed through a 2-mm sieve. The soil samples were preincubated at the field water holding capacity for one week at ambient temperature (25 ± 0.2 °C).

### Preparation of labelled winter wheat straw

Uniformly ^13^C-labelled winter wheat straw (δ^13^C straw = 13,070‰) was obtained by growing wheat using ^13^C-labelled carbon dioxide (^13^CO_2_)-C in a closed growth system that consisted of an upper chamber made of Plexiglas and a bottom chamber made of PVC pipe. The upper chamber was used for labelling and had a volume of 25.12 L (20-cm diameter × 80-cm height). Two holes were located in the top and bottom walls of the upper chamber. The bottom hole was used for the injection of HCl (which reacted with the Ba^13^CO_3_ inside the chamber to produce ^13^CO_2_), and the top hole was connected with a transparent 500-mL plastic bag to buffer the pressure shift inside the chamber. The bottom chamber (20-cm diameter × 30-cm height) was used for the soil.

The upper and bottom chambers were connected by a PVC lid and fixed with screws. There were two holes in the upper walls of the bottom chamber and one hole in the PVC lid. The chamber holes were used for watering and the lid hole for the plants to grow through. To prevent gas leakage, Vaseline was applied to all of the interfaces between each part and rubber plugs were applied to seal the holes.

The labelling was conducted from 9:00 am to 17:00 every day. The chamber was moved to a low-temperature laboratory at noon to reduce the inside temperature. The transpiration water was drawn by injection in the evening if necessary. The amount of Ba^13^CO_3_ (enrichment >98 atom ^13^C%, Shanghai Research Institute of Chemical Industry) used was 30 g for the two chambers. After 30 d of labelling, the aboveground part of the plant was oven-dried and ground (<1 mm) before its addition to the soil samples. The straw and soil samples (60 g soil) were thoroughly mixed at a rate of 5 g straw kg^−1^ soil^26^,^27^.

### Incubation experiment

**Figure 1:**
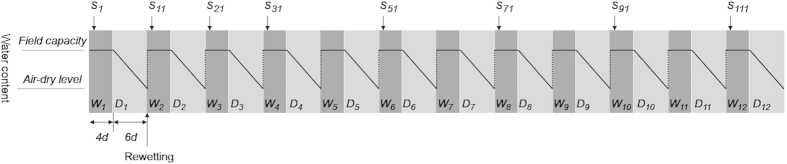
Sequence of treatment and water content during drying and wetting (DW) cycles. W, D and S represent wetting (4 d), drying (6 d), and sampling (first day after rewetting), respectively, during each DW cycle. One complete DW cycle included 4 d wetting and 6 d air-drying. Field capacity was maintained constant during the 4 d wetting incubations.

Daily precipitation records for the area from the same period (1991 to 2010) for the area were used to determine the duration of the wetting and drying cycle for the experiment. The data showed that the soils experienced dry conditions 60% of the year, thus a wetting and drying cycle consisted of 6 days of dry conditions and 4 days of wet conditions. Twelve DW cycles were implemented during the experimental period (120 d). The treatments and their combinations were as follows (2 straw treatments × 2 tillage-treated soils × 2 water contents): (i) with or without straw input, (ii) soil from the CT field or NT field and (iii) with or without exposure to DW cycles, which was characterised by wetting at 100% WHC (water-holding capacity,) and air-drying (10–15% WHC) at 25 °C in an incubator with controlled temperature and humidity (Fig. 1).

The treatments without DW cycles were constantly kept at field capacity during the experiment by adding deionised water^26^,^28^. Each experimental unit was incubated in a 400-mL glass jar. The glass jars were flushed with CO_2_ free air and sealed with a greased rubber ring and a lid with a septum for the needle. Three replicates for each treatment were prepared.

### Measurements of CO_2_ concentration and δ^13^C

The organic carbon mineralisation was monitored by measuring the CO_2_ and the δ^13^C-CO_2_ at days 1, 2, 11, 21, 31, 51, 71, 91 and 111. The CO_2_ that was released during the drying periods was not measured, as this study focuses on the carbon mineralisation dynamics rather than the carbon budget. The CO_2_ concentration was determined by taking a 5-mL sample with a 10-mL syringe. The headspace was mixed 10 to 15 times before sampling with a 100-mL syringe^3^. The used bottles were vacuumised with ultra-high purity He, and a volume equal to the expected sample volume was added to the 20-mL bottle to maintain the atmospheric pressure. The CO_2_ concentration (μmol mol^−1^) was measured on an HP5890 gas chromatograph (Agilent Technologies Inc., Palo Alto, CA, USA), and the carbon isotope ratios were measured on a trace gas system interfaced with an IsoPrime mass spectrometer (IsoPrime Ltd., Cheadle Hulme, UK). At the beginning of the experiment and after each CO_2_ measurement, the atmosphere of the jar was free of CO_2_. The carbonate contents and δ^13^C of the CO_2_ from carbonate in the soil samples were measured at the beginning and end of the incubation period to assess the extent of CO_2_ efflux and exchange from carbonate.

### δ^13^C calculations and PE quantification

The ^13^C isotopic composition of the CO_2_, straw and soil samples was expressed in δ units (‰)^29^. The standard equation for determining δ^13^C (‰) is derived from


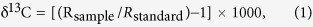


where R = ^13^C/^12^C. The δ^13^C values were calculated with reference to Vienna Pee Dee Belemnite (R_standard _= ^13^C/^12^C = 0.0112372). Therefore, Equation 1 can be rearranged to solve for the isotopic ratio of samples as follows:


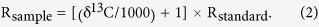


The ^13^C atom percentage (%) of the sample is


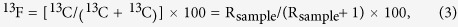


where ^13^F (%) is the ^13^C fraction of CO_2_ in the atmosphere of the sample. To distinguish between the different sources of the respired CO_2_, the mass balance of ^13^C was applied as follows^30^,^31^:


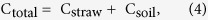


where C_total_, C_straw_ and C_soil_ represent the total CO_2_ that was captured from each jar, from the straw and from the soil, respectively. In terms of isotopes, Equation 4 can be rearranged in terms of ^13^C as follows:


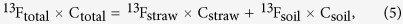


where ^13^F_total_, ^13^F_straw_ and ^13^F_soil_ represent the ^13^C atom % of CO_2_ in the atmosphere of each jar, of the straw and of the soil. The contribution of CO_2_ originating from soil (C_soil_) decomposition was calculated as


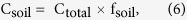


where f_soil_ is the proportion of evolved CO_2_ from the soil. The contribution of CO_2_ originating from straw (C_straw_) decomposition was calculated as


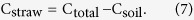


Therefore, Equation 8 can be obtained as follows:


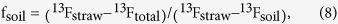


where ^13^F_soil_ can be replaced with the ^13^C atom % of CO_2_ in the atmosphere of the control experiment.

The PE that was induced by the winter wheat straw was calculated by comparing the amount of ^12^CO_2_ in the samples with the winter wheat straw to the amount of ^12^CO_2_ in the control treatments. The PE intensity was calculated according to the following equation^14^:


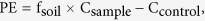


where C is the amount of CO_2_ in the jar’s headspace (sample or control) in mg C-CO_2_ kg^−1^ dry soil. Consequently, the PE is here considered as the difference between the SOC mineralisation with straw and the SOC mineralisation without straw. In this study, native soil-derived C represents the CO_2_ that evolved from the soil with the winter wheat straw^30^,^32^.

### Statistical analyses

All of the statistical analyses were performed using Microsoft Excel 2007 (Microsoft Corporation, USA) and SPSS Windows^®^ version 18.0 (SPSS Inc., Chicago, USA). We conducted analyses of variance (ANOVA) in repeated measures with replicates as error terms to analyse the effects of the DW cycles and winter wheat straw on the SOC decomposition rate at different sampling times. The least significant difference (LSD at *P* < 0.05) test was performed to assess the differences among the means of three replicates of these variables.

## Results

### Effects of DW cycles on the rate of SOC mineralisation

**Figure 2:**
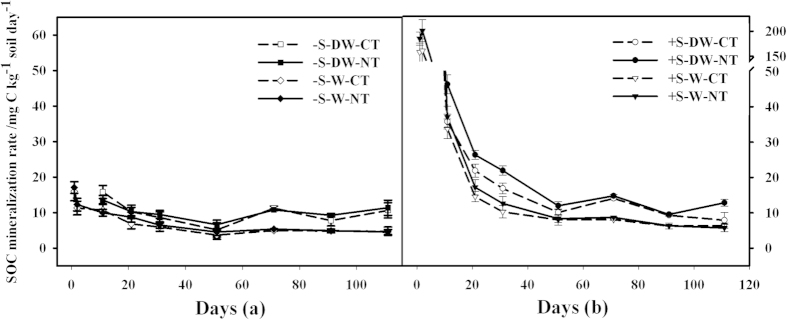
Rates of soil organic carbon (SOC) mineralisation for the no-tillage (NT) or conventional-tillage (CT) soils amended with (+S) or without (−S) wheat straw in the drying and wetting (DW) cycles or continuously wet (W) treatments. Values are expressed in mg of C-CO_2_ kg^−1^ of dried soil day^−1^. Error bars represent the standard error of the means (similarly hereinafter).

After rewetting, the rate of SOC mineralisation in the DW cycle treatment was significantly (*P* < 0.01) greater than that in the continuously wet treatment (Fig. 2), with an average of 3.90 mg C-CO_2_ kg^−1^ soil day^−1^ during the whole incubation period. Additionally, the SOC mineralisation fluctuated greatly after the 5th cycle. In the continuously wet treatment, the rate of organic carbon mineralisation gradually decreased with incubation time: it sharply decreased on day two, moderately decreased for a period (2 d–50 d) and finally remained stable after day 51. During the incubation, the rate of SOC mineralisation varied alternatively between the CT soils and the NT soils, with an average of 9.97 and 7.71 mg C-CO_2_ kg^−1^ soil day^−1^ for the CT soils and 10.22 and 8.94 mg C-CO_2_ kg^−1^ soil day^−1^ for the NT soils under DW cycles and continuously wet treatments, respectively, with no significant (P > 0.05) difference.

After the straw input, the rate of CO_2_ efflux in the amended soils (+S) was significantly (*P* < 0.01) higher than that in the unamended soils (−S). The rate on day two of the continuously wet treatment was slightly accelerated and then sharply decreased from 2 d-11 d by 142.80 mg C-CO_2_ kg^−1^ soil day^−1^. After the 1st cycle (10 d), the mineralisation rate in both the DW cycles and the continuously wet treatments slowed down gradually with the incubation time. However, the mineralisation rate in the DW cycle treatment was, on average, 5.46 mg C-CO_2_ kg^−1^ soil day^−1^ greater than that in the continuously wet treatment. The difference in CO_2_ efflux rate between the CT and NT treatments was significant for 11 d–31 d, and there was no significant difference during most of the whole incubation. The interaction effects between the straw, water condition and soil tillage management were not significant (*P* > 0.05) (Table 2).

### Proportion of CO_2_ from straw in the soil with straw input

**Figure 3:**
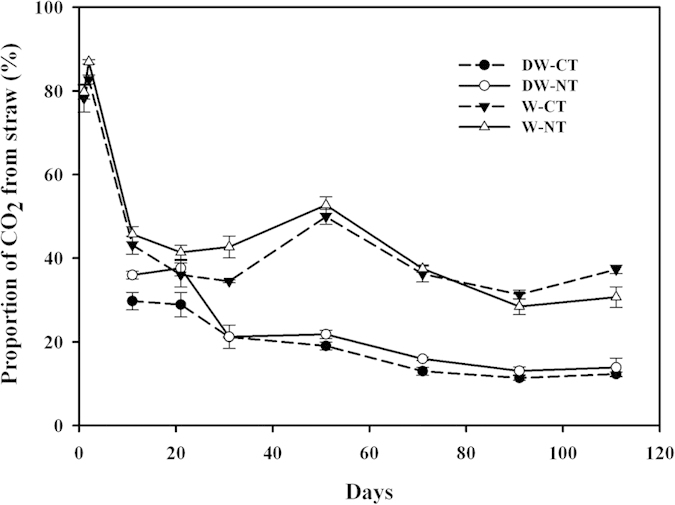
Proportion of CO_2_ from straw in the no-tillage (NT) or conventional-tillage (CT) soils amended with winter wheat straw in the drying and wetting (DW) cycles or continuously wet (W) treatment.

In the continuously wet treatment, the proportion of CO_2_ from the straw in the soils that were amended with straw first increased and then decreased, while in the DW cycles, it decreased gradually (Fig. 3). In the DW cycles, the proportion for the NT was greater than that of the CT, with no significant difference (*P* > 0.05). In the early stage of incubation (before 71 d), the mean proportion of CO_2_ from straw for the NT was only 3.72% greater than that of the CT in the continuously wet treatment, while in the later stage (91 d–111 d), the results were reversed, still with no significant difference (*P* > 0.05). From the rewetting day, the proportion of CO_2_ from the straw varied from 44% to 34% in the continuously wet treatment and was significantly (*P* < 0.001) greater than that in the DW cycles which varied from 33% to 13%. The interaction effects between the water condition and soil tillage management were not statistically significant (*P* > 0.05).

### PE during incubation

**Figure 4:**
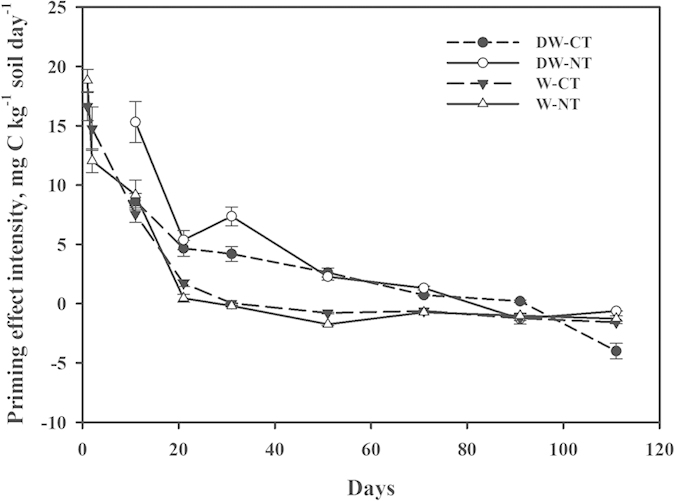
Priming effect (PE) intensity for the no-tillage (NT) or conventional-tillage (CT) soils amended with winter wheat straw in the drying and wetting (DW) cycles or continuously wet (W) treatments. Values are expressed in mg of C-CO_2_ kg^−1^ of dried soil day^−1^.

The PE intensity gradually decreased with the incubation time and acted first positively and then slightly negatively under the DW cycles and continuously wet treatments (Fig. 4). A negative PE occurred after 31 d in the continuously wet treatment and after 91 d in the DW cycles. The PE intensity of the no-tillage and conventional-tillage soils in the DW cycles was greater than that in the continuously wet treatment, with an average of 1.71 and 3.58 mg C-CO_2_ kg^−1^ soil day^−1^ respectively. Specifically, the difference in the PE between the DW cycles the and constant moisture conditions for 11 d–71 d was significant (*P* < 0.05); this difference was not significant (*P* > 0.05) after 91 d. In contrast with the soils without straw input in the continuously wet treatment, the DW cycles and straw together significantly increased the rate of SOC mineralisation demonstrating a positive combined effect. In the first five DW cycles and during the first 11 days in the continuously wet treatment, the straw had a greater influence on the rate of SOC mineralisation than did the DW cycles by 3.45 and 4.01 mg C kg^−1^ soil day^−1^, respectively; subsequently, the straw had less influence by 5.88 and 3.82 mg C kg^−1^ soil day^−1^, respectively (Figs 2 and 4). During the incubation period, the differences in PE between the conventional-tillage (CT) soils and the NT soils were not significant (*P* > 0.05), but the interaction between the water condition and soil tillage management was significant (*P* < 0.05).

## Discussion

### Effect of DW cycles on the rate of C mineralisation

Dry-wet cycles increased C mineralisation by approximately 50% compared with a continuously moist soil. This result agreed with some studies that indicated that DW could accelerate organic matter decomposition and increase SOC mineralisation^6^,^33^,^34^ but disagreed with others^3^,^35^. Most of those contrasting results can be explained by the different frequency & severity of the Dry-Wet cycles to which the soils are subjected to. That is, the DW frequency/length can determine not only the extent, but even the direction of the effects on CO_2_ effluxes and SOC decomposition.

The rate of total CO_2_ efflux of the straw input in the continuously wet treatment first increased and then sharply decreased, while it gradually decreased in the DW treatment, generally confirming the results of Cosentino *et al.*^28^ and Zhang *et al.*^36^. This study indicated that, in contrast with the continuously wet treatment, the rate of CO_2_ efflux from the soils and straw in the DW cycles was greater, which was attributed to the acceleration in the rate of soil native organic carbon mineralisation despite the decrease in the straw decomposition rate^8^, which was also verified by the PE in this study.

### Effect of straw on the C mineralisation rate

The rate of CO_2_ efflux under the straw input treatments substantially increased compared to the treatments without straw input, which agreed with the results of Mamilov *et al.* (2002)^37^. After the first ten days, the mineralised carbon from the straw significantly decreased with incubation time. Under the two water conditions, the PE of the straw was first positive, gradually decreased and then became slightly negative. Our results suggest that straw addition accelerated soil organic matter mineralisation. The mechanisms of PE remain elusive, but triggering microbial biomass activity to consume additional substrates is an important component^38^. Fontaine *et al.*^39^ reported that cellulose stimulated at least two types of microorganisms, one that exclusively decomposes cellulose and another that mostly decomposes SOM but may also use cellulose. It is therefore plausible that the addition of the wheat straw stimulated the SOM decomposing microorganisms; however, the stimulatory effect was limited and temporary, as these organisms were gradually outcompeted by other microorganisms utilising the straw^40^, which caused the decreasing PE associated with CO_2_ production.

### Priming effects under the DW cycles

Our results indicate that drying-wetting cycles can impact PE. This study provides evidence that DW cycles exert significant control on PE and thus CO_2_ efflux soils. Our study also shows that the PE under drying-wetting conditions is higher than that under constantly wet conditions. This effect of drying-wetting on soil C dynamics through PE should be further explored and be incorporated into ecosystem models to improve the prediction of the response of soil carbon storage. This study also shows that for PE the interaction is often significant, which suggests that the effect of water treatment varies with tillage treatment (Table 2). However, we see that the effect of water treatment is overwhelming (Fig. 4). In fact, even if statistically significant, the interaction effect is probably very small. For example, for each water treatment, the two tillage treatments are very close to one another.

The PE of the straw was greater in the DW cycles than in the continuously wet treatment, presenting an interaction between the soil water regime and the straw addition. Winter wheat straw had a greater influence on the rate of SOC mineralisation than did the DW cycles during early incubation, while in the later stage, the reverse was observed. In the early stage, the large straw input stimulated microbial activities, while in the later stage, the amount gradually reduced, thereby decreasing the influence of the straw. These results differed somewhat from the study of Zhang *et al.*^36^, who concluded that rice straw had a greater influence on SOC mineralisation than did the DW cycles, based on the fact that the respiration rate of soils increased 2.6 fold due to rice straw input under continuous wetting and decreased 12–39% due to the DW cycles compared to continuous wetting (both with straw input). This study, however, determined the influence of the straw based on its PE under the continuously wet treatment or the DW cycles. We studied the differences between the DW cycles and the continuously wet treatment without straw input (excluding the interaction between the straw and moisture). Our study may provide interesting models for studying the operation of PE.

### Differences between no-tillage and conventional-tillage systems

Our study indicated that the major factor controlling PE intensity are straw and DW cycles but not tillage management. The difference in the SOC mineralisation rate and PE intensity between the NT and CT soils was not significant. In comparison, Paulis^41^ in Ontario, Canada, found no significant effect of tillage management on SOC mineralisation rate in medium- and heavy-textured soils following the addition of C-labeled soybean residues. A long-term NT and CT study also showed no significant effect of the tillage system on the SOC mineralisation rate^20^,^42^. The interaction between the water condition and soil tillage management did not affect the SOC mineralisation rate either (Table 2) but significantly affected the PE intensity with straw input (Fig. 4). The DW cycles and straw incorporation significantly increased the rate of SOC mineralization, demonstrating a positive combined effect^26^. Based on this, it is assumed that the comparison of tillage system effects on the respiration of CO_2_ and ^13^CO_2_ was influenced by the drying and wetting cycles. Future work on this topic should focus on structurally intact soils.

## Conclusions

Drying and wetting cycles have a great effect on C mineralisation and on PE. The SOC mineralisation rate in rewetted soils was greater than that in soils that were kept at constant water content. The PE of the straw was greater in the DW cycles than in the continuously wet treatment. The intensity of the PE that was induced by wheat straw decreased with incubation time. The priming direction of the wheat straw was first positive and then became slightly negative. We conclude that, after the addition of straw, environmental changes including fluctuations in the soil moisture and SOC contents would influence the direction and intensity of the PE. Furthermore, winter wheat straw had a greater influence on the rate of SOC mineralisation than did the DW cycles during early incubation, while in the later stage the case was reversed. The priming was mainly controlled by the DW cycles but not the tillage. However, further research is still needed to focus on the soil microbial community and C pools to understand how they alter the PE of DW cycles.

## Additional Information

**How to cite this article**: Liu, E. *et al.* Priming effect of ^13^C-labelled wheat straw in no-tillage soil under drying and wetting cycles in the Loess Plateau of China. *Sci. Rep.* **5**, 13826; doi: 10.1038/srep13826 (2015).

## Figures and Tables

**Table 1:** Properties of soil samples (0–20 cm) and ^13^C-labelled winter wheat. Values represent the means of three replicates with the standard deviation (SD) in parentheses.

Soil parameter	CT^a^	NT	labelled winter wheat straw
pH(H_2_O)	7.5 (0.1)	7.4 (0.2)	—
Organic carbon (g kg^−1^)	6.43 (0.11)	7.44 (0.12)	422.10 (0.51)
Organic nitrogen (g kg^−1^)	0.40 (0.02)	0.63 (0.03)	5.00 (0.07)
Clay content (<2 μm) (%)	15.8 (0.9)	16.3 (1.4)	—
Microbial biomass carbon (mg kg^−1^)	121 (12)	255 (14)	—
Particulate organic carbon(53–2000 μm) (g kg^−1^)	1.8 (0.2)	2.8 (0.3)	—
Dissolved organic carbon (mg kg^−1^)	30 (4)	45 (6)	—
Calcium carbonate (%)	8.94 (0.04)	8.37 (0.04)	—
Electric conductivity (μS cm^−1^)	120.48 (0.46)	122.60 (0.60)	—
Mean weight diameter (mm)	0.28 (0.07)	0.60 (0.07)	—

^a^CT: conventional-tillage, NT: no-tillage.

**Table 2:** Results of analysis of variance with the factors of straw, water condition and soil tillage management.

Sampling time (d)	1	2	11	21	31	51	71	91	111	Mean
Rate of soil organic carbon mineralisation										
S^a^	***^b^	***	***	***	***	***	***	***	NS	***
W			***	***	***	**	***	***	***	***
T	NS	NS	**	**	**	NS	NS	NS	NS	NS
S × W			NS	***	***	NS	NS	NS	NS	NS
S × T	NS	NS	***	NS	*	NS	*	NS	NS	*
W × T			NS	NS	NS	NS	NS	NS	*	NS
S × W × T			*	NS	NS	NS	NS	NS	NS	NS
Proportion of CO_2_ from straw										
W			***	*	***	***	***	***	***	***
T	NS	*	NS	**	*	NS	NS	NS	NS	NS
W × T			NS	NS	*	NS	NS	*	**	NS
Priming effect intensity										
W			***	***	***	***	***	***	***	***
T	NS	NS	***	NS	**	NS	NS	NS	***	NS
W × T			**	*	*	*	*	**	***	**

^a^S, W and T represent straw, water condition and soil tillage management.

^b^*, **, ***Indicate significance at *P* < 0.05, 0.01 and 0.001, respectively.
